# Eye and Ear Infirmary. or Oculist and Aurist

**Published:** 1879-04

**Authors:** 


### "Eye and Ear Infirmary," or "Oculist and Aurist."

The Medical Society of the State of New York, at the Annual Meeting held June 18th, 1878, passed the following resolution—the subject being presented by the legal proceedings recently instituted in the Niagara County Medical Society:

WHEREAS, The question has been referred to us for decision. Now, therefore, for the purpose of declaring an authoritative decision upon the question, and for the information of the profession, therefore be it

*Resolved*, That it is a breach of the Code of Medical Ethics of this Society for a member of a County Society, entitled to send delegates to this Society, to use upon signs, bill heads or advertisements, by way of advertising his profession, the words 'Eye and Ear Infirmary,' or 'Oculist and Aurist.'

